# Alterations in lipid transfers to HDL associated with the presence of coronary artery disease in patients with type 2 diabetes mellitus

**DOI:** 10.1186/s12933-015-0270-8

**Published:** 2015-08-14

**Authors:** Marilia C O Sprandel, Whady A Hueb, Alexandre Segre, José A F Ramires, Roberto Kalil-Filho, Raul C Maranhão

**Affiliations:** Lipid Metabolism Laboratory, Heart Institute, Medical School Hospital, University of São Paulo, São Paulo, Brazil; Clinical Cardiology Division, Heart Institute, Medical School Hospital, University of São Paulo, São Paulo, Brazil; Faculty of Pharmaceutical Sciences, University of São Paulo, São Paulo, Brazil

**Keywords:** Coronary artery disease, Type 2 diabetes, Lipid transfers, High-density lipoprotein, HDL, Nanoparticles, Cholesterol

## Abstract

**Background:**

We previously showed that unesterified-cholesterol transfer to high-density lipoprotein (HDL), a crucial step in cholesterol esterification and role in reverse cholesterol transport, was diminished in non-diabetic patients with coronary artery disease (CAD). The aim was to investigate whether, in patients with type 2 diabetes mellitus (T2DM), the occurrence of CAD was also associated with alterations in lipid transfers and other parameters of plasma lipid metabolism.

**Methods:**

Seventy-nine T2DM with CAD and 76 T2DM without CAD, confirmed by cineangiography, paired for sex, age (40–80 years), BMI and without statin use, were studied. In vitro transfer of four lipids to HDL was performed by incubating plasma of each patient with a donor emulsion containing radioactive lipids during 1 h at 37 °C. Lipids transferred to HDL were measured after chemical precipitation of non-HDL fractions and the emulsion. Results are expressed as % of total radioactivity of each lipid in HDL.

**Results:**

In T2DM + CAD, LDL-cholesterol and apo B were higher than in T2DM. T2DM + CAD also showed diminished transfer to HDL of unesterified cholesterol (T2DM + CAD = 7.6 ± 1.2; T2DM = 8.2 ± 1.5 %, p < 0.01) and of cholesteryl-esters (4.0 ± 0.6 vs 4.3 ± 0.7, p < 0.01). Unesterified cholesterol in the non-HDL serum fraction was higher in T2DM + CAD (0.93 ± 0.20 vs 0.85 ± 0.15, p = 0.02) and CETP concentration was diminished (2.1 ± 1.0 vs 2.5 ± 1.1, p = 0.02). Lecithin-cholesterol acyltransferase activity, HDL size and lipid composition were equal.

**Conclusion:**

Reduction in T2DM + CAD of cholesterol transfer to HDL may impair cholesterol esterification and reverse cholesterol transport and altogether with simultaneous increased plasma unesterified cholesterol may facilitate CAD development in T2DM.

## Background

The most frequent fatal complication of type 2 diabetes mellitus (T2DM) is coronary artery disease (CAD), accounting for nearly 60 % of deaths among T2DM patients. Patients with T2DM are exposed to a two-four fold higher risk of developing CAD than the general population [1]. Thus, it is of fundamental importance in T2DM management to determine the factors that predispose these patients to develop CAD. Several steps in the metabolism of the plasma lipoproteins are regulated by insulin [2] and in T2DM the appearance of dyslipidemia, characterized by hypertriglyceridemia and low high-density lipoprotein (HDL) cholesterol are frequent and may increase CAD risk [3–5].

In respect to HDL, the negative correlation between HDL-cholesterol and the incidence of CAD was well established in several epidemiologic studies [6–8]. However, it has been perceived that this lipoprotein, that has several protective anti-atherogenic functions, should not be evaluated only by HDL-cholesterol concentration but also by methods testing the functional aspects.

In the bloodstream, HDL is constantly being remodeled and transfer of lipids from other lipoproteins and from the peripheral cells to HDL and from HDL to other lipoprotein classes is crucial in the HDL metabolism. Nascent HDL is produced in the liver and intestine as discoid particles composed of apolipoprotein (apo) A-I, phospholipids and unesterified cholesterol [9]. These particles are progressively transformed into a spherical shape by the transfer of lipids to HDL and cholesterol esterification. HDL is thus continuously being remodeled, and lipid transfers to and from the lipoprotein are an essential part of HDL metabolism and atheroprotective functions [10]. Transfer of lipids is facilitated by transfer proteins such as cholesteryl ester transfer protein (CETP) and phospholipid transfer protein (PLTP) that are involved in the transfers of core (cholesteryl esters and triglycerides) and surface (phospholipids and unesterified cholesterol) lipids, respectively [11]. The role of CETP and PLTP in atherogenesis and T2DM is still not completely understood [12, 13], but the HDL status is clearly more affected by lipid transfers than VLDL and LDL. Impairment of lipid transfers by CETP inhibitors leads to pronounced HDL increases, with lesser effects on the other lipoprotein classes [14]. CETP inhibition aiming to increase HDL cholesterol levels has become a novel target in anti-atherogenic therapeutics [14], and this highlights the importance to investigate transfer of lipids involving HDL.

Cholesterol esterification, a reaction that is catalyzed by lecithin-cholesterol acyltransferase (LCAT), using apo A-I as a co-factor [9], is essential for stabilization of the cholesterol plasma pool and occurs mainly in the HDL fraction that contains both LCAT and apo A-I. In the reverse cholesterol transport process, HDL shuttles cholesterol from peripheral tissues to the liver for excretion in the bile. This process is not affected by the increased serum levels of advanced glycation end-products (AGE) occurring in T2DM [15]. HDL has also anti-inflammatory, anti-oxidant, anti-coagulant and anti-apoptotic properties, all presumably protective against CAD [9].

Recently, we described an in vitro assay to assess the simultaneous transfer to HDL of the four main lipids, unesterified cholesterol, cholesteryl esters, phospholipids, and triglycerides, from a donor lipid nanoemulsion to HDL [16, 17]. We subsequently reported that the lipid transfers were disturbed in patients with premature CAD. In those patients, among other changes in lipid transfers, the transfer of unesterified cholesterol to the HDL fraction was diminished, independent of the HDL-cholesterol levels [18].

It is fundamental to explore the features of T2DM patients with concomitant CAD that distinguishes this population from T2DM patients that did not develop the disease. Understanding of those features may improve the prevention of the macrovascular complications. Thus, the aim of this study was to investigate whether development of CAD in T2DM patients is associated with alterations in lipid transfers to HDL, as compared with T2DM patients without CAD. Factors related with this process, such as HDL lipid composition, plasma CETP, LCAT and unesterified cholesterol were also studied in the two groups.

## Methods

### Study subjects

For this case–control study, patients with T2DM were selected from the Outpatient Clinic of the Heart Institute at the University of São Paulo Medical School. All participated of the MASS study group [19]. Patients that were using statins or other lipid-lowering drugs discontinued this treatment for at least 45 days before they participated in this study. Seventy-nine patients had cardiovascular disease confirmed by coronary cineangiography showing lesions with >50 % luminal reduction (T2DM + CAD), whereas 76 patients had angiographically normal arteries (T2DM). Exclusion criteria were severe hypertension (based on the *Joint National Committee VI criteria for* blood pressure >180/110 mmHg), renal failure (when plasma creatinine levels were above the reference threshold), hepatic impairment (if activity of aspartate aminotransferase, alanine aminotransferase, gamma-glutamyltransferase, and alkaline phosphatase were above the reference thresholds), hypothyroidism and recent surgery. The two groups were paired for sex, age, and BMI.

The study conformed to the guidelines set out in the Declaration of Helsinki and was approved by the Ethics Committee of the University of São Paulo Medical School. All participants gave written informed consent for participation in the study.

### Laboratory analysis

Plasma total cholesterol (CHOD-PAP; Roche, Basel, Swiss) and triglycerides (Triglyceride Rapid; Roche) were determined by using commercial kits (Labtest, Minas Gerais, Brazil) from plasma samples collected after a 12-h fast. HDL-cholesterol was measured by the same method used for total cholesterol after lipoprotein precipitation with magnesium phosphotungstate. LDL-cholesterol was estimated by the Friedewald formula [20]. The non-HDL cholesterol level was calculated by subtracting the HDL from the total cholesterol concentration. Unesterified cholesterol and non-esterified fatty acids (NEFA) were also determined by an enzymatic colorimetric method (Wako, Richmond, VA, USA). Serum concentrations of apo A-I and apo B were determined by the immunoturbidimetric method (Roche, Mannhein, Germany).

### HDL particle size determination

The diameter of the HDL fraction particles was measured in fresh plasma collected after a 12-h fast as described previously [21], using a ZetaPALS Zeta Potential Analyzer (Brookhaven, Holtsville, NY, USA).

### Lipid composition of the HDL fraction

The HDL fraction was obtained from the whole plasma after precipitation of the apo B-containing lipoproteins with magnesium phosphotungstate. Triglyceride concentration in the HDL fraction was determined using a commercial kit (Labtest, Minas Gerais, Brazil). Unesterified cholesterol and phospholipid concentrations were determined by using an enzyme-linked immunosorbent assay (Wako, Richmond, VA, USA). Esterified cholesterol in the HDL fraction was calculated as the difference between total and unesterified cholesterol of the HDL multiplied by 1.67 to adjust for M.W. of esterified cholesterol [22].

### Nanoemulsion preparation

The lipid donor nanoemulsion was prepared from a lipid mixture composed of 40 mg cholesteryl oleate, 20 mg egg phosphatidylcholine, 1 mg triolein, and 0.5 mg cholesterol purchased from Sigma Chemical Company (St. Louis, MO, USA). Emulsification of lipids by prolonged ultrasonic irradiation in aqueous media and the procedure of two-step ultracentrifugation of the crude emulsion with density adjustment by addition of KBr to obtain the nanoemulsion was carried out by the method described previously [16]. The nanoemulsion fraction was dialyzed against 0.9 % NaCl solution. Trace amounts of cholesteryl [-^14^C] oleate and glycerol tri [9, 10(n)-^3^H] oleate or [7(n)-^3^H] cholesterol and l-3-phosphatidylcholine, 1-stearoyl-2-[1-^14^C] arachidonyl (Perkin Elmer, Waltham, MA, USA) were added to the initial solution.

### Lipid transfer from the donor lipid nanoemulsion to HDL

The in vitro assay of lipid transfer from the nanoemulsion to HDL was previously described by Lo Prete et al. [16]. In brief, it consisted of the incubation of the nanoemulsion labeled with ^3^H-Cholesteryl ester and ^14^C-Phospholipid or with ^14^C-Unesterified cholesterol and ^3^H-Triglyceride with whole plasma followed by chemical precipitation of the apo B containing lipoproteins and the nanoemulsion. After the chemical precipitation of the nanoemulsion and the apo B lipoprotein fraction, the supernatant containing the HDL fraction radioactivity was measured in a scintillation solution. The results of radioactive transfer from the nanoemulsion to HDL were expressed as percentage of the total incubated radioactivity found in the HDL-containing supernatant.

### Determination of CETP concentration and LCAT activity

Serum CETP activity was measured using commercially available CETP activity assay kit (BioVision, Mountain View, CA, USA). Serum CETP concentration was determined by the immunoassay method  (ALPCO Diagnostics, Salem, NH, USA) and plasma LCAT activity was determined by fluorometric assay kit (Calbiochem, Germany). This assay is based on the hydrolysis of an artificial LCAT substrate that fluoresces at 470 nm, resulting in a product that fluoresces at 390 nm. LCAT activity is defined as the change in the ratio of 390/470 nm fluorescence emission intensities.

### Statistical analysis

The results are expressed as mean ± SD. Data were compared using the unpaired Student *t* test. The Fisher’s exact test was used to analyze percentages. Pearson’s test was used for correlation analyses. In all analyses, parameters were considered significantly different when p < 0.05. Multiple linear regression analysis was used to determine the independent contribution of variables to lipid transfers. The model was based on variables that had significant association with lipid transfers on univariate analyses, besides age, sex, BMI and CAD presence. Statistical analyses were performed using SPSS 20.0 for Windows (SPSS, Chicago, IL, USA).

## Results

Table 1 shows the physical and clinical data of the study subjects. The two groups were different only in time elapsed after diabetes diagnosis, but were equal regarding age, BMI, waist circumference, and percentage of subjects with arterial hypertension. T2DM and T2DM + CAD groups were also equal regarding percentage taking insulin and the diabetes control parameters fasting glucose and HbA_1c_.

**Table 1:** Clinical and physical characteristics of T2DM patients with or without CAD

Parameters	T2DM	T2DM + CAD	*P* value
Number of patients (M/F)	75 (33/42)	79 (42/37)	NS
Age (years)	63 ± 9	63 ± 8	NS
BMI (kg/m^2^)	31.4 ± 6.1	30.8 ± 6.0	NS
Waist circumference (cm)	107 ± 16	104 ± 13	NS
Time from T2DM diagnosis (years)	7 ± 6	10 ± 9	0.01
Arterial hypertension (%)	93	89	NS
Fasting glucose (mmol/L)	7.3 ± 1.8	7.0 ± 2.4	NS
HbA_1c_ % (mmol/L)	7.2 ± 1.6 (55 ± 17)	7.1 ± 1.8 (54 ± 19)	NS
Current medications (%)			
Insulin	25	29	NS
Metformin	67	84	0.02
Sulfonylurea	29	25	NS
ACEi	58	43	NS
Beta-blocker	63	76	NS

Data are expressed as mean ± SD.

*NS* nonstatistically significant, *M* Male, *F* Female, *BMI* body mass index, *HbA*_*1c*_ glycated hemoglobin, *ACEi* angiotensin converting enzyme inhibitor.

Serum biochemical parameters of the two groups are shown in Table 2. Regarding the two main parameters involved in diabetes dyslipidemia, namely triglycerides and HDL-cholesterol were not different in the two groups. In contrast, LDL (p < 0.0001) and non-HDL-cholesterol (p = 0.01) were higher in the T2DM + CAD than in the T2DM group. Serum unesterified cholesterol levels were also higher in T2DM + CAD than in T2DM (p = 0.02). Regarding the serum concentrations of the apos, apo B was higher in T2DM + CAD (p = 0.03), whereas apo A-I did not differ between the two groups. The triglycerides/HDL cholesterol and LDL cholesterol/apo B ratios were not different between the two groups. Table 3 shows the transfer of the four lipids from the donor nanoemulsion to the HDL plasma fraction. The transfers of unesterified cholesterol and of cholesteryl ester were diminished in the T2DM + CAD patients compared to the T2DM group (p < 0.01), but the phospholipid and triglyceride transfer rates were similar. Also shown in Table 3, when comparing the two groups, the lipid composition of the HDL fraction and the HDL size were not different.

**Table 2:** Serum biochemistry data of T2DM patients with or without CAD

Parameters	T2DM	T2DM + CAD	*P* value
Triglycerides (mmol/L)	1.72 ± 0.78	1.91 ± 0.81	NS
Cholesterol (mmol/L)			
Total	4.99 ± 0.93	5.63 ± 1.24	<0.001
LDL	3.20 ± 0.85	3.80 ± 1.13	<0.001
HDL	1.06 ± 0.23	0.98 ± 0.20	NS
Non-HDL	3.90 ± 0.95	4.65 ± 1.18	0.01
Unesterified cholesterol	0.85 ± 0.15	0.93 ± 0.20	0.02
Triglycerides/HDL-cholesterol	8.5 ± 5.1	10.4 ± 7.3	NS
Apolipoproteins (mg/dL)			
A-I	139.3 ± 28.1	132.5 ± 24.3	NS
B	96.0 ± 19.5	103.1 ± 20.4	0.03
LDL cholesterol/apo B	1.34 ± 0.29	1.23 ± 0.57	NS
NEFA (g/L)	0.13 ± 0.06	0.11 ± 0.05	NS
Total/unesterified cholesterol	5.8 ± 1.0	6.2 ± 1.2	0.05
CETP (µg/mL)	2.5 ± 1.1	2.1 ± 1.0	0.02
CETP activity (pmol/µL/h)	68.4 ± 15.6	70.4 ± 12.5	NS
LCAT activity	1.33 ± 0.10	1.34 ± 0.12	NS

Data are expressed as mean ± SD.

*NS* nonstatistically significant,* HDL* high density lipoprotein, *LDL* low density lipoprotein, *NEFA* non-esterified fatty acids, *CETP* cholesteryl ester transfer protein, *LCAT* lecithin-cholesterol acyltransferase, *LCAT* activity is expressed by the hydrolyzed monomer ratio 390/470 nm.

**Table 3:** In vitro lipid transfers to HDL and HDL composition in T2DM patients with or without CAD

Parameters	T2DM	T2DM + CAD	*P* value
Lipid transfers (%)			
Unesterified cholesterol	8.2 ± 1.5	7.6 ± 1.2	0.007
Esterified Cholesterol	4.3 ± 0.7	4.0 ± 0.6	0.005
Phospholipids	25.5 ± 2.6	25.9 ± 2.1	NS
Triglycerides	5.0 ± 0.7	4.9 ± 0.8	NS
Lipid composition of HDL (%)			
Unesterified cholesterol	6.0 ± 1.7	5.6 ± 1.6	NS
Esterified cholesterol	34.7 ± 5.6	34.0 ± 5.5	NS
Phospholipids	50.3 ± 6.1	52.0 ± 6.0	NS
Triglycerides	8.9 ± 2.5	8.4 ± 2.6	NS
HDL diameter (nm)	9.0 ± 0.6	8.9 ± 1.2	NS

Data are expressed as mean ± SD.

*NS* nonstatistically significant, *HDL* high density lipoprotein.

The concentration of CETP in the serum was lower in T2DM + CAD than in T2DM patients (p = 0.02), but CETP activity was not different between groups. The activity of the cholesterol esterification enzyme LCAT was also similar in the two groups (Table 2).

Table 4 shows the results of the correlation study performed by univariate analysis in the two groups studied (T2DM and T2DM + CAD). Values of the transfer of the four lipids, CETP serum concentration, LCAT activity and serum concentration of unesterified cholesterol were plotted against the several parameters measured here. Significant positive correlations were found between transfers of all four lipids and HDL-cholesterol and apo A-I, whereas negative correlations were found between the transfers of the four lipids and LCAT activity.

**Table 4:** Correlations between lipid transfers to HDL, serum concentrations of CETP, unesterified cholesterol and LCAT activity

Parameters	Lipid transfers				Unesterified cholesterol	LCAT activity	CETP concentration
	Unesterified cholesterol	Cholesteryl ester	Phospholipid	Triglyceride			
HDL-cholesterol	0.633^†^	0.333^†^	0.269^†^	0.383^†^	−0.088	−0.313^†^	0.168
LDL-cholesterol	−0.005	0.218*	0.220^†^	0.182*	0.416^†^	−0.170	0.059
Tryglicerides	−0.229^†^	0.103	−0.144	0.030	0.381^†^	0.034	−0.012
non HDL-cholesterol	−0.078	0.212*	0.188*	0.180*	0.511^†^	−0.120	0.050
TG/HDL-cholesterol	−0.322^†^	0.046	−0.059	−0.044	0.403^†^	0.083	−0.020
Unesterified cholesterol	−0.255^†^	0.119	0.007	0.040	–	−0.044	0.058
Total/unesterified cholesterol	0.226^†^	0.128	0.253^†^	0.154	−0.402^†^	−0.059	0.033
NEFA	0.490	0.044	−0.158	−0.054	0.003	0.265^†^	−0.056
Fasting glucose	−0.154	0.081	−0.098	−0.108	0.148	0.100	0.018
HbA1_c_	−0.022	0.027	−0.054	0.044	0.166*	−0.037	0.068
Apo A-I	0.697^†^	0.584^†^	0.316^†^	0.334^†^	−0.039	−0.383^†^	0.305^†^
Apo B	−0.219^†^	0.091	0.109	0.096	0.617^†^	−0.141	0.096
CETP concentration	0.185*	0.522^†^	0.117	0.183*	0.058	−0.149	0.032
LCAT activity	−0.460^†^	−0.235^†^	−0.317^†^	−0.283^†^	−0.044	–	−0.056
Cholesteryl ester transfer	0.364^†^	–	0.048	0.224^†^	0.119	−0.235^†^	0.522^†^
Phospholipid transfer	0.568^†^	0.048	–	0.576^†^	0.007	−0.317^†^	0.117
Unesterified cholesterol transfer	–	0.364^†^	0.568^†^	0.561^†^	−0.255^†^	−0.460^†^	0.185*
Triglyceride transfer	0.561^†^	0.224^†^	0.576^†^	–	0.040	−0.283^†^	0.183*
HDL diameter	0.055	0.122	−0.099	−0.006	−0.150	0.037	0.100
HDL lipid composition							
Unesterified cholesterol	0.321^†^	0.121	0.057	0.204*	−0.218*	−0.111	0.181*
Tryglicerides	−0.134	0.185*	−0.151	−0.193*	−0.004	0.135	0.196*
Phospholipids	−0.161	−0.035	−0.036	−0.129	0.123	0.037	−0.176
Cholesteryl ester	0.141	−0.084	0.091	0.170	−0.066	−0.066	0.049

*BMI* Body mass index, *HDL* high density lipoprotein, *LDL* low density lipoprotein, *TG* triglycerides, *UC* unesterified cholesterol, *HbA1*_*c*_ glycated hemoglobin, *Apo* apolipoprotein, *CETP* cholesteryl ester transfer protein, *LCAT* lecithin-cholesterol acyltransferase, *NEFA* non-esterified fatty acids.

* p value <0.05.

^†^p value <0.01.

The unesterified cholesterol transfer to HDL positively correlated with the serum concentration of triglycerides and with the percentage of unesterified cholesterol in the composition of the HDL fraction. Unesterified cholesterol transfer to HDL correlated negatively with apo B, serum concentration of unesterified cholesterol and with the ratio triglycerides/HDL-cholesterol and positively with CETP concentration. The cholesteryl ester transfer to HDL was positively correlated with LDL-cholesterol, triglyceride content in HDL and CETP concentration. The phospholipid transfer to HDL correlated positively with LDL-cholesterol. The triglyceride transfer to HDL correlated positively with LDL-cholesterol and non HDL-cholesterol concentrations. The triglyceride transfer also positively correlated with the percentage of unesterified and the percentage of esterified cholesterol in the composition of the HDL fraction and with the serum concentration of CETP. All the transfer rates positively correlated with each other, except the transfers of cholesteryl ester and phospholipids.

The serum concentration of unesterified cholesterol correlated positively with serum concentration of LDL-cholesterol, triglycerides, non-HDL-cholesterol and apo B. The serum concentration of unesterified cholesterol negatively correlated with the percentage of unesterified cholesterol in the composition of the HDL fraction.

Cholesteryl ester transfer protein concentration in the serum correlated positively with the concentrations of triglycerides and of apo A-I, as well as with the percentage of triglycerides and the percentage of unesterified cholesterol in the composition of the HDL fraction. LCAT activity positively correlated with serum concentration of NEFA and negatively with the concentration of HDL-cholesterol and of apo A-I.

By multiple linear regression analysis, unesterified cholesterol transfer was independently associated with the presence of CAD (β = −0.416, p = 0.003), LCAT activity (β = −3.184, p < 0.001), apo A-I (β = −0.037, p < 0.001), apo B (β = −0.017, p < 0.001), fasting glycemia (β = −0.005, p = 0.003), and sex (β = −0.365, p = 0.021).

Cholesteryl ester transfer to HDL was independently associated with the presence of CAD (β = −0.211, p = 0.031), CETP concentration (β = 0.241, p < 0.001), HDL-cholesterol (β = −0.02, p = 0.009), LDL-cholesterol (β = 0.003, p = 0.024), and apo A-I (β = 0.017, p < 0.001).

Phospholipid transfer to HDL was independently associated with LDL-cholesterol (β = 0.009, p < 0.001), apo A-I (β = 0.022, p ≤ 0.001), age (β = −0.026, p = 0.003), sex (β = −0.403, p = 0.023), but not with CAD (p = 0.796).

Triglyceride transfer to HDL was independently associated with LCAT (β = −1.2, p = 0.05) and apo A-I (β = 0.009, p = 0.001), but not with CAD (β = −0.139, p = 0.273).

T2DM + CAD had total and LDL cholesterol and apo B concentrations greater than in T2DM, which could influence the results of lipid transfers. To address this issue, an analysis was made by pairing the groups according to their values of total, LDL and HDL cholesterol and apo B. In this analysis, T2DM + CAD had 64 and T2DM had 65 participants. After this pairing, the outcome of the results remained the same, i.e., compared to T2DM, T2DM + CAD had diminished transfer of unesterified (7.6 ± 1.2 vs 8.2 ± 1.5 %, p = 0.01) and esterified cholesterol (3.9 ± 0.6 vs 4.3 ± 0.7 %, p < 0.01) whereas triglyceride (4.9 ± 0.8 vs 5.0 ± 0.8 %, NS) and phospholipid (25.9 ± 2.1 vs 25.7 ± 2.6 %, NS) transfers were equal.

## Discussion

The results of this study, in which all participants had the presence or absence of CAD confirmed by coronary cineangiography, indicate that the plasma lipid atherogenic alterations classically associated with T2DM, i.e., low HDL-cholesterol and hypertriglyceridemia, did not discriminate between T2DM and T2DM + CAD patients. The triglycerides/HDL-cholesterol ratio which is also related with insulin resistance and atherogenesis [23, 24] was also not discriminative between the T2DM and T2DM + CAD groups. Rather, higher LDL-cholesterol, non-HDL-cholesterol and apo B were the lipid markers for T2DM + CAD. The ratio LDL-cholesterol/apo B, which is a surrogate for the more atherogenic small dense LDL sub-fraction, and is altered in T2DM was not different between the two groups.

Data in the literature comparing the plasma lipid profile of T2DM patients with and without CAD are scarce. In this respect, similar to our findings, Seviour et al. [25] reported that LDL-cholesterol and apo B concentrations were higher in T2DM with CAD than in T2DM patients without CAD, without differences in HDL-cholesterol or triglyceride values. In the study by Kahri et al. [26], all three plasma lipid parameters, LDL and HDL-cholesterol and triglycerides, were equal in T2DM with or without CAD. Thus, the two previous and the current study converge in the finding that low HDL-cholesterol and high triglycerides, were not differentiating factors between CAD and non-CAD T2DM patients. Likewise, NEFA, which is characteristically elevated in T2DM resulting from the mobilization of fatty acids in insulin action deficiency, were not different between the two groups. This is a first-time observation to our knowledge, NEFA was not examined as a CAD risk factor in T2DM patients in either transversal, as the current study, or in prospective studies from the literature.

The main novel finding in this study was the diminished transfer of unesterified cholesterol to the HDL fraction in T2DM + CAD, despite the fact that the HDL-cholesterol and apo A-I concentrations were equal in both groups. Diminished transfer of unesterified cholesterol was also found in patients with precocious (<50 years) CAD without T2DM in comparison with non-CAD control subjects. In that study, all analyzed traditional lipid risk factors such as LDL and HDL-cholesterol and triglycerides were equal in CAD and non-CAD subjects [18].

In T2DM + CAD unesterified cholesterol concentration in total and in non-HDL plasma fraction was higher than in T2DM. In contrast, in the HDL plasma fraction the unesterified cholesterol concentration was equal in the two groups. The association of higher unesterified cholesterol serum concentration with CAD without T2DM was reported by Frohlich and Dobiasova in women [27], but our current results in T2DM + CAD are a novel finding.

In the correlation study, we found that the unesterified cholesterol serum concentration correlates negatively with unesterified cholesterol transfer to HDL and unesterified cholesterol in HDL composition. The suggestion is then left that because less unesterified cholesterol enters in the HDL wherein it is esterified, more remains in the non-HDL fraction.

The activity of LCAT, the enzyme that catalyzes cholesterol esterification in the plasma, did not differ between T2DM + CAD and T2DM, but was negatively correlated with all lipid transfers. The status of LCAT activity is a controversial issue in atherosclerosis [28–30] and has not yet been investigated in T2DM with CAD. On the other hand, the fact that esterified cholesterol transfer was negatively correlated with LCAT activity suggests that higher rates of cholesterol esterification tend to stabilize the HDL fraction, so that the entry of all lipids into the lipoprotein particles is inhibited.

Esterification stabilizes the cholesterol pool because newly esterified cholesterol is sequestered into the lipoprotein core. Unesterified cholesterol at the lipoprotein surface can conceivably diffuse into the surrounding aqueous medium and eventually precipitate in the artery. In contrast, cholesteryl esters at the lipoprotein core are translocated from the lipoprotein only by a CETP-mediated process [31]. In addition, cholesterol esterification is also a driving force for the reverse transport, since it creates the gradient for the surface of the HDL particles to receive additional unesterified cholesterol from the cells. Diminution of unesterified cholesterol transfer to HDL can then diminish the esterification process by diminishing the substrate offer, and impairing the cholesterol reverse transport.

In a previous study, we injected lipidic nanoemulsions with labeled unesterified cholesterol and cholesteryl ester in CAD and control non-CAD subjects, all with presence or absence of CAD documented by coronary cineangiography: the removal of unesterified cholesterol from the plasma was faster than that of cholesteryl ester in CAD, indicating that in CAD patients unesterified cholesterol was being dissociated from the nanoparticles [32]. The possibility was raised that unesterified cholesterol dissociated from the particles could be deposited in the arterial wall, as suggested by analyzing vessel fragments of patients who underwent revascularization previously injected with the labeled nanoemulsion [31].

Thus, in CAD patients, regardless of their having T2DM or not, the suggestion is left from the lipid transfer assay results and unesterified cholesterol serum concentration that the presence of CAD is associated with dynamic aspects of unesterified cholesterol intravascular metabolism. It would be tempting to examine whether the transfer of unesterified cholesterol from cultivated murine macrophages to HDL would also be decreased in T2DM + CAD, as Khera et al. showed in non-diabetic CAD patients [33]. Similarly to our previous study in non-diabetic CAD patients, our results of diminished transfer of unesterified cholesterol to HDL in T2DM + CAD also point to a second defective step in unesterified cholesterol metabolism, now involving the entry of unesterified cholesterol from lipoproteins in the HDL fraction. Indeed, the ability of HDL to accept unesterified cholesterol is essential for cholesterol esterification and drives reverse cholesterol transport.

Another outcome of the lipid transfer study was the decrease in the transfer of cholesteryl ester to HDL in T2DM + CAD. Since CETP facilitates cholesteryl ester transfers, this was probably related to the diminution of the concentration of CETP observed in T2DM + CAD. In T2DM, CETP concentration was reportedly high in two studies [34, 35] and low in one [36]. The current study is the first to compare T2DM patients without and with CAD. In this context, it could be postulated that high CETP concentration, together with high cholesteryl ester transfer to HDL is protective against CAD in T2DM patients. Phospholipid and triglyceride transfers were not altered in T2DM + CAD relative to T2DM. In respect to triglycerides, since CETP concentration was decreased in T2DM + CAD it would be expected that the triglyceride entry would also be decreased, since triglyceride transfers are also mediated by CETP and, as expected, in our correlation study, decreased CETP concentration corresponded to decreased triglyceride transfer to HDL.

Cholesteryl ester transfer protein also positively correlated with the triglyceride content in HDL composition. This is quite expected because the net result of CETP action is enrichment of the HDL fraction with triglycerides.

It is worthwhile to point out that many factors present in the plasma that are difficult to account for can interfere in the lipid transfers to HDL. In this respect, Morton et al. [37] reported that the unesterified cholesterol content of the donor lipoproteins could modulate cholesteryl ester transfer without changing triglyceride transfer. Since the unesterified cholesterol pool in the non-HDL is increased in T2DM + CAD, it is possible that the donor nanoemulsion becomes unesterified cholesterol-enriched in the plasma of those patients, which would contribute for the results of T2DM + CAD vs T2DM.

The lipid composition of the HDL fraction did not differ between T2DM + CAD and T2DM, which is somewhat unexpected in view of the decreased transfers to HDL of both unesterified cholesterol and cholesteryl ester. However, lipid transfers are bidirectional and the transfer assay used here measures only the entry of lipids into the HDL fraction and not the lipid exit. Thus, the fact that the unesterified cholesterol or the cholesteryl ester transfers were diminished does not necessarily imply a reduction of those lipids in HDL composition.

As limitations of the current study, some of the great number of variables that are present in T2DM, such as time from diabetes diagnosis, anti-glycemic and anti-hypertensive medications, as well as lifestyle interventions, were not equal in the two groups and may have eventually influenced the results.

An important finding of the correlation analysis was that the transfer of all four lipids positively correlated with the HDL-cholesterol and with the apo A-I concentration. This reflects the mass effect observed in the methodological study on lipid transfers [16], such that the greater the HDL concentration, expressed by the HDL-cholesterol and apo A-I concentrations, the higher the transfer of lipids to the HDL fraction.

## Conclusion

This study shows that although lower HDL-cholesterol was not a marker for the presence of CAD in patients with T2DM, the cholesterol transfers to the lipoprotein, both in the esterified and in the non-esterified forms, were indeed altered in T2DM + CAD patients as compared to T2DM. Since HDL is the main cholesterol esterification site in the plasma compartment, decrease in the transfer of unesterified cholesterol to this lipoprotein fraction can interfere with the esterification process, which is critical for the stabilization of the plasma cholesterol pool and the reverse cholesterol transport. In this regard, the unesterified cholesterol concentration in the plasma of the T2DM + CAD was higher than in T2DM, which is an atherogenic factor and can be accounted for the diminished transfer to HDL. In respect to the observed reduction in cholesteryl ester transfer to HDL in these patients, it was probably related with the lower CETP levels found in the T2DM + CAD group. These results highlight the importance of the systematic dynamic studies of HDL metabolic and functional aspects beyond the simple determination of lipoprotein plasmatic concentration. As exemplified in this study, those novel parameters can unravel novel atherogenic mechanisms and therapeutic targets.

## Abbreviations

CAD: coronary artery disease
T2DM: type 2 diabetes mellitus
LCAT: lecithin-cholesterol acyltransferase
APO: apolipoprotein
CETP: cholesteryl ester transfer protein
PLTP: phospholipid transfer protein
NEFA: non-esterified fatty acids
AGE: advanced glycation end-products

## References

[1] Pasterkamp G (2013). Methods of accelerated atherosclerosis in diabetic patients. Heart.

[2] Leança CC, Nunes VS, Panzoldo NB, Zago VS, Parra ES, Cazita PM (2013). Metabolism of plasma cholesterol and lipoprotein parameters are related to a higher degree of insulin sensitivity in high HDL-C healthy normal weight subjects. Cardiovasc Diabetol.

[3] Mooradian AD (2009). Dyslipidemia in type 2 diabetes mellitus. Nat Clin Pract Endocrinol Metab.

[4] Fruchart JC, Davignon J, Hermans MP, Al-Rubeaan K, Amarenco P, Assmann G (2014). Residual macrovascular risk in 2013: what have we learned?. Cardiovasc Diabetol.

[5] Yassine HN, Belopolskaya A, Schall C, Stump CS, Lau SS, Reaven PD (2014). Enhanced cholesterol efflux to HDL through the ABCA1 transporter in hypertriglyceridemia of type 2 diabetes. Metabolism.

[6] Gordon T, Castelli WP, Hjortland MC, Kannel WB, Dawber TR (1977). High density lipoprotein as a protective factor against coronary heart disease. The Framingham Study. Am J Med.

[7] Gordon DJ, Probstfield JL, Garrison RJ, Neaton JD, Castelli WP, Knoke JD (1989). High-density lipoprotein cholesterol and cardiovascular disease. Four prospective American studies. Circulation.

[8] Acharjee S, Boden WE, Hartigan PM, Teo KK, Maron DJ, Sedlis SP (2013). Low Levels of high density lipoprotein cholesterol and increased risk of cardiovascular events in stable ischemic heart disease patients: a post hoc analysis from the COURAGE Trial. J Am Coll Cardiol.

[9] Kontush A, Chapman MJ (2006). Functionally defective high-density lipoprotein: a newtherapeutic target at the crossroads of dyslipidemia, inflammation, and atherosclerosis. Pharmacol Rev.

[10] Rosenson RS, Brewer HB, Davidson WS, Fayad ZA, Fuster V, Goldstein J (2012). Cholesterol efflux and atheroprotection: advancing the concept of reverse cholesterol transport. Circulation.

[11] Tall A (1995). Plasma lipid transfer proteins. Ann Rev Biochem.

[12] Siebel AL, Natoli AK, Yap FY, Carey AL, Reddy-Luthmoodoo M, Sviridov D (2013). Effects of high-density lipoprotein elevation with cholesteryl ester transfer protein inhibition on insulin secretion. Circ Res.

[13] Abbasi A, Dallinga-Thie GM, Dullaart RP (2015). Phospholipid transfer protein activity and incident type 2 diabetes mellitus. Clin Chim Acta.

[14] Barter PJ, Rye KA (2012). Cholesteryl ester transfer protein inhibition as a strategy to reduce cardiovascular risk. J Lipid Res.

[15] Low H, Hoang A, Forbes J, Thomas M, Lyons JG, Nestel P (2012). Advanced glycation end-products (AGEs) and functionality of reverse cholesterol transport in patients with type 2 diabetes and in mouse models. Diabetologia.

[16] Lo Prete AC, Dina CH, Azevedo CH, Puk CG, Lopes NH, Hueb WA (2009). In vitro simultaneous transfer of lipids to HDL in coronary artery disease and in statin treatment. Lipids.

[17] Maranhao RC, Freitas FR (2014). HDL metabolism and atheroprotection: predictive value of lipid transfers. Adv Clin Chem.

[18] Maranhao RC, Freitas FR, Strunz CM, Santos RD, Mansur AJ, Mansur AP (2012). Lipid transfers to HDL are predictors of precocious clinical coronary heart disease. Clin Chim Acta.

[19] Hueb WA, Bellotti G, de Oliveira SA, Arie S, de Albuquerque CP, Jatene AD (1995). The Medicine, Angioplasty or Surgery Study (MASS): a prospective, randomized trial of medical therapy, balloon angioplasty or bypass surgery for single proximal left anterior descending artery stenoses. J Am Coll Cardiol.

[20] Friedewald WT, Levy RI, Fredrickson DS (1972). Estimation of the concentration of low-density lipoprotein cholesterol in plasma, without use of the preparative ultracentrifuge. Clin Chem.

[21] Lima E, Maranhão RC (2004). Rapid, simple laser-light scattering method for HDL particle size in whole plasma. Clin Chem.

[22] Bragdon JH, Eder HA, Gould RG, Havel RJ (1956). Lipid nomenclature: recommendations regarding the reporting of serum lipids and lipoproteins made by the Committee on Lipid and Lipoprotein Nomenclature of the American Society for the Study of Arteriosclerosis. Circ Res.

[23] Marotta T, Russo BF, Ferrara LA (2010). Triglyceride-to-HDL-cholesterol ratio and metabolic syndrome as contributors to cardiovascular risk in overweight patients. Obesity.

[24] Hermans MP, Ahn SA, Rousseau MF (2014). Novel unbiased equations to calculate triglyceride-rich lipoprotein cholesterol from routine non-fasting lipids. Cardiovasc Diabetol.

[25] Seviour PW, Teal TK, Richmond W, Elkeles RS (1988). Serum lipids, lipoproteins and macrovascular disease in non-insulin-dependent diabetics: a possible new approach to prevention. Diabet Med.

[26] Kahri J, Syvanne M, Taskinen MR (1994). Plasma cholesteryl ester transfer protein activity in non-insulin-dependent diabetic patients with and without coronary artery disease. Metabolism.

[27] Frohlich J, Dobiasova M (2003). Fractional esterification rate of cholesterol and ratio of triglycerides to HDL-cholesterol are powerful predictors of positive findings on coronary angiography. Clin Chem.

[28] Ng DS (2012). The role of lecithin: cholesterol acyltransferase in the modulation of cardiometabolic risks—a clinical update and emerging insights from animal models. Biochim Biophys Acta.

[29] Savel J, Lafitte M, Pucheu Y, Pradeau V, Tabarin A, Couffinhal T (2012). Very low levels of HDL cholesterol and atherosclerosis, a variable relationship—a review of LCAT deficiency. Vasc Health Risk Manag.

[30] Rousset X, Shamburek R, Vaisman B, Amar M, Remaley AT (2011). Lecithin cholesterol acyltransferase: an anti- or pro-atherogenic factor?. Curr Atheroscler Rep.

[31] Couto RD, Dallan LA, Lisboa LA, Mesquita CH, Vinagre CG, Maranhao RC (2007). Deposition of free cholesterol in the blood vessels of patients with coronary artery disease: a possible novel mechanism for atherogenesis. Lipids.

[32] Santos RD, Hueb W, Oliveira AA, Ramires JA, Maranhao RC (2003). Plasma kinetics of a cholesterol-rich emulsion in subjects with or without coronary artery disease. J Lipid Res.

[33] Khera AV, Cuchel M, de la Llera-Moya M, Rodrigues A, Burke MF, Jafri K (2011). Cholesterol efflux capacity, high-density lipoprotein function, and atherosclerosis. New Eng J Med.

[34] Triolo M, Kwakernaak AJ, Perton FG, de Vries R, Dallinga-Thie GM, Dullaart RP (2013). Low normal thyroid function enhances plasma cholesteryl ester transfer in type 2 diabetes mellitus. Atherosclerosis.

[35] Dullaart RP, de Vries R, Dallinga-Thie GM, van Tol A, Sluiter WJ (2007). Plasma cholesteryl ester transfer protein mass and phospholipid transfer protein activity are associated with leptin in type 2 diabetes mellitus. Biochim Biophys Acta.

[36] Inukai Y, Ito K, Hara K, Yamazaki A, Takebayashi K, Aso Y (2007). Serum cholesteryl ester transfer protein concentrations are associated with serum levels of total cholesterol, beta-lipoprotein and apoproteins in patients with type 2 diabetes mellitus. Med Princ Pract..

[37] Morton RE (1988). Free cholesterol is a potent regulator of lipid transfer protein function. J Biol Chem.

